# Effect of light‐cured pulp capping materials on human dental pulp cells in vitro

**DOI:** 10.1111/iej.14242

**Published:** 2025-04-25

**Authors:** Laurentia Schuster, Sonja Sielker, Johannes Kleinheinz, Till Dammaschke

**Affiliations:** ^1^ Department of Periodontology and Operative Dentistry University of Münster Münster Germany; ^2^ Clinic for Oral and Maxillofacial Surgery University Hospital Münster Münster Germany

**Keywords:** biodentine, light‐curing pulp capping materials, pulp capping, vital pulp therapy

## Abstract

**Aim:**

Gold standard as material in vital pulp therapy (VPT) is hydraulic calcium silicate cements (HCSC). To circumvent their prolonged setting time, light‐cured pulp capping materials containing calcium silicate or calcium hydroxide powder are available. Although their positive biological properties are advertised, the data regarding the biocompatibility of light‐cured pulp capping materials (LCPCM) is inconclusive. This in vitro study compared the biocompatibility of five LCPCM containing calcium silicate (TheraCal LC, ReviCal, MTA PulpCap, Pulprotec MTA) or calcium hydroxide (Calcimol LC) with that of the HCSC Biodentine.

**Methods:**

Of each material, 226 cylindrical specimens (51.472 mm^3^) were prepared and incubated in a sterile cell culture medium (alpha Modified Eagle Medium) for 24 h to obtain an extract. Human dental pulp cells (hDPC) were added to the specimens and/or extracts. Cell viability and changes in cell morphology were examined (MTT, LDH, live‐dead staining, light microscope). Calcium release from the materials (Ca^2+^ colorimetric assay) and the mineralisation capacity of the cells (Alizarin Red S Staining, Alkaline Phosphatase Assay) were determined. Statistical analysis was performed by anova and the post‐hoc Tukey test (*p* < 0.05).

**Results:**

Compared to Biodentine, hDPC showed significantly lower cell viability when in contact with LCPCM (*p* < 0.05). Further, an inhibition zone around the test bodies or an altered cell morphology was observed. Biodentine showed almost no negative effects on cell viability or cell morphology. In contact with Biodentine, hDPC mineralise with and without mineralisation induction conditions. Among the LCPCM, mineralisation was only detectable under induction conditions with ReViCal and MTA PulpCap. In addition, Biodentine released significantly more calcium ions than the LCPCM (*p* < 0.05).

**Conclusion:**

In this in vitro study, LCPCM showed cytotoxic effects on hDPC and were hardly able to induce cell mineralisation. Biodentine showed little negative effects on cell viability, induced cell mineralisation and released more calcium than LCPCM. Biodentine is significantly superior to LCPCM in terms of biocompatibility and mineralisation induction capacity.

## INTRODUCTION

With the correct clinical indication and the selection of a suitable pulp capping material, vital pulp therapy (VPT) can have high clinical success rates—regardless of the type of pulp exposure (carious exposure or trauma) (Dammaschke et al., 2025; Harms et al., 2019; Wang et al., 2017). For successful VPT, the pulp capping material used should induce new hard tissue formation (bioactivity) and exhibit no toxic effects on the pulp tissue (biocompatibility). It should seal the pulpal wound from other (possibly toxic) restoration materials and the oral cavity, thus preventing microbial infection of the pulp.

Under this aspect, hydraulic calcium silicate cements (HCSC) like ProRoot MTA have proven to be suitable pulp capping materials (Dammaschke et al., 2014; Dammaschke et al., 2025).

Nevertheless, all available calcium silicate cements do share the disadvantage of a relatively long setting time, which can range from approximately 15 min up to several hours (About, 2022; Kaup et al., 2015). Hence, in recent years, several light‐cured flowable composite resins containing calcium hydroxide or calcium silicate powder have been introduced to avoid the disadvantage of the long waiting time for setting. The manufacturers of these light‐cured pulp capping materials (LCPCM) promise to offer a fast‐setting pulp capping material that combines the advantages of a composite resin with those of calcium hydroxide or HCSC. However, the results of in vitro and in vivo studies that can be found in the literature regarding the biocompatibility of LCPCM are heterogeneous (Bakhtiar et al., 2017; Hebling et al., 2009; Manaspon et al., 2021; Petrolo et al., 2014; Sahin et al., 2021; Sanz et al., 2021). For some materials, even no data can be found in the literature. Some authors report high clinical success rates comparable to HCSC (Cannon et al., 2014; Erfanparast et al., 2018; Gurcan & Seymen, 2019). Nevertheless, histological examinations of the pulp reporting favourable outcomes when using LCPCM for VPT are lacking. In contrast, relevant studies have proven that LCPCM release a steady amount of monomers and other components (Küden et al., 2022; Nilsen et al., 2017), which exhibit cytotoxic effects on cells (Hanks et al., 1991; Issa et al., 2004) and can cause an inflammatory reaction of the pulp tissue (Bakhtiar et al., 2017; Lee et al., 2015). Thus, further investigations into the biological properties of LCPCM need to be conducted for a better understanding of their biocompatibility and to determine their suitability as pulp capping material in comparison to HCSC or calcium hydroxide.

The aim of the present study was to assess the biological properties of five different LCPCM in comparison to the HCSC Biodentine by examining their effects on human dental pulp cells in vitro. For the following LCPCM analysed here up to now, no data have been found in the literature: ReViCal (R‐Dental, Hamburg, Germany), MTA PulpCap (Cumdente, Tübingen, Germany) and Pulprotec MTA (Cumdente, Tübingen, Germany). Therefore, this is the first time that these materials have ever been analysed in terms of their biocompatibility.

## MATERIALS AND METHODS

The manuscript of this laboratory study has been written according to the Preferred Reporting Items for Laboratory studies in Endodontology (PRILE) 2021 guidelines (Nagendrababu et al., 2021), as depicted in the PRILE flowchart.
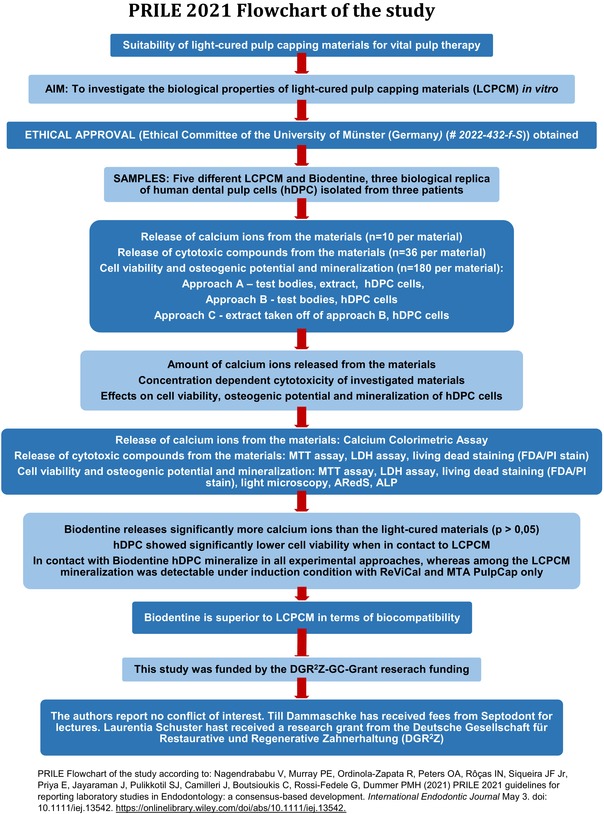


### Pulp capping materials

The following pulp capping materials were selected (Table 1): Biodentine (BD; Septodont, Saint‐Maur‐des‐Fossés, France), ReViCal (RVC; R‐Dental, Hamburg, Germany), TheraCal LC (TLC; BISCO, Schaumburg, IL, USA), MTA PulpCap and Pulprotec MTA (MPC and PPM; both Cumdente, Tübingen, Germany) and Calcimol LC (CLC; VOCO, Cuxhaven, Germany).

**TABLE 1:** Pulp capping materials investigated, ingredients according to the manufacturers' SDS.

Product name	Material	Ingredients	Manufacturer	Indications
Biodentine	Hydraulic calcium silicate cement Powder and liquid	Powder: tricalcium silicate, zirconium oxide, calcium oxide, calcium carbonate Liquid: aqueous calcium chloride and polycarboxylate	Septodont, Saint‐Maur‐des‐Fossés, France	Pulpotomy, direct and indirect pulp capping
Calcimol LC	Flowable composite resin 1‐component‐system Light‐curing	Calcium hydroxide, fumed silica, UDMA, TEGDMA	VOCO, Cuxhaven, Germany	Indirect pulp capping
TheraCal LC	Flowable composite resin 1‐component‐system Light‐curing	Portland cement type III, fumed silica, Bis‐GMA, polyglycol dimethacrylate, barium zirconate	BISCO, Schaumburg, IL, USA	Direct and indirect pulp capping
ReViCal	Flowable composite resin 1‐component‐system Light‐curing	Mixture of various mineral oxides and methacrylates: Mineral oxides GHS05, GHS07, H315, H317, H318; Methacrylates GHS07, H315, H319, H335, H317	R‐dental, Hamburg, Germany	Direct and indirect pulp capping
MTA PulpCap	Flowable composite resin 1‐component‐system Light‐curing	Mixture of various mineral oxides and methacrylates: Mineral oxides GHS05, GHS07, H315, H318, H317; Methacrylates GHS07, H315, H319, H335, H317	Cumdente, Tübingen, Germany	Direct and indirect pulp capping
Pulprotec MTA	Flowable composite resin 1‐component‐system Light‐curing	Mixture of various mineral oxides and methacrylates: Mineral oxides GHS05, GHS07, H315, H318, H317; Methacrylates GHS07, H315, H319, H335, H317	Cumdente, Tübingen, Germany	Indirect pulp capping

All materials were used directly without prior treatment. The materials were pressed into moulds (6.4 mm diameter; 1.2 mm height; 51.472 mm^3^ volume), covered from both sides with glass plates and light cured according to the manufacturer's protocols for 20 s (CLC and TLC) and 40 s (RVC, MPC and PPM), respectively, from both sides of the moulds (to ensure complete polymerisation) with a LED light‐curing device at 1000 mW/m^2^ and 460–490 nm wavelength (SmartLite Focus, Dentsply Sirona, Charlotte, NC, USA). Biodentine was mixed according to the manufacturer's protocol, pressed into moulds (see above), covered from both sides with glass plates and left to set for 15 min. All test bodies were sterilised with ultraviolet light and thereafter maintained in sterile conditions. In total, 226 samples per material were produced.

### Cell culture

The isolation and cultivation of human dental pulp cells (hDPC) were performed according to the protocol established by Sabandal et al. (2020). All cell samples were taken after the patients' informed consent. Ethical approval for the use of human cells was obtained by the Ethical Committee of the University of Münster (Germany) (# 2022‐432‐f‐S). The handling of all human samples followed strictly the guidelines set forth in the Declaration of Helsinki. For this study, the effects on three isolated cell cultures as biological replicates were analysed and summarised. All assays described in the following sections were performed in triplicate.

Cells were cultivated in alpha Modified Eagle Medium (aMEM) supplemented with 10% fetal bovine serum (FBS), 1% amphotericin B [250 mg/mL] and 1% penicillin [10.000 U/mL]/streptomycin [10 mg/mL] (all PAN‐Biotech, Aidenbach, Germany). Cells were cultivated at 37°C in a humidified atmosphere with 5% CO_2_ (standard condition). The culturing medium was replaced twice a week, and the cells were passaged after reaching 90% confluence. For the induction of mineralisation, 16 ng/mL dexamethasone (Fortecortin, Merck Pharma, Darmstadt, Germany), ascorbic acid [1.4 mM] and ß‐glycerophosphate [10 mM] were added additionally (all Merck/Sigma‐Aldrich, Darmstadt, Germany).

### Cell viability and osteogenic differentiation

Test bodies were put in 24‐well culture plates (Greiner Bio‐one, Frickenhausen, Germany) in three different experimental approaches (A, B and C; see Table 2). For approach A, test bodies were put in culture plates, then the cell culture medium (alpha Modified Eagle Medium, see the ‘Cell culture’ section) was added in a defined volume (1 mL) to the materials for 24 h under sterile conditions. The culture medium, now called extract, as well as the test bodies remained in the wells for consecutive steps. For approach B, the extract was taken off after 24 h and the same volume of fresh cell culture medium (1 mL) was added to the test bodies. For approach C, the extract (1 mL) taken off from approach B was used. In approaches A and B, the test bodies remained in the well during the whole study. For approach C, only extract and no test body was used. Cells were seeded with a defined density (20 000/cm^2^) to approaches A, B and C. The extract remained for another 48 h with the cells and was replaced with a fresh culturing medium afterward. Cells were cultivated for up to 14 days with and without a mineralisation inducing medium. As a control group, cells with no contact to the test bodies or extract were used. Cell culture was performed with three technical replicates (see ‘Cell culture’ section).

**TABLE 2:** Main experimental design used for cell viability, osteogenic potential and mineralisation examinations.

	Approach A	Approach B	Approach C
Extract	Remains in well	Was exchanged by freshly added cell culture medium	Extract taken off from approach B was used
Material	Test body remains in well	Test body remains in well	No material itself
Experimental conditions	Cells are exposed to the material and the extract and subsequently fresh cell culture medium	Cells are exposed to the material and fresh cell culture medium	Cells are exposed to the material extract and subsequently fresh cell culture medium

### Release of calcium ions

The release of calcium ions from the materials was determined using a calcium detection kit (Calcium Colorimetric Assay, Merck/Sigma‐Aldrich, Darmstadt, Germany). Sterilised test bodies were produced as described above and put in 48‐well culturing plates (Greiner Bio‐one, Frickenhausen, Germany) and culturing medium (without FBS or glutamine) with a defined volume (1 mL per well) was added. Samples were kept under standard conditions to gain reproducibility with cell culture conditions. Samples were collected at 24 h, 48 h, 7 days and 14 days. After that, the medium was diluted to the defined starting volume to maintain a constant concentration. As a control, a culturing medium without test bodies was used. Ten technical replicates were used for each material. Samples were stored at −20°C until analysed.

### Effects of pulp capping materials' extracts on cell viability in different dilutions

Thirty‐six test bodies of each tested material were produced as described above. Cell culturing medium in a defined volume (see ‘Cell culture’ section) was added to the materials for 24 h in order to obtain an extract. Extracts were taken, afterward sterile filtered and stored at −20°C until use. Cells were seeded with a defined density (20 000/cm^2^) in 48‐well culturing plates (Greiner Bio‐one, Frickenhausen, Germany) and allowed to adhere for 24 h. Supplements, such as FBS, antibiotics and antimycotics, were added to the extracts (see ‘Cell culture’ section). A serial dilution of the extracts of each tested material was performed (0%, 10%, 25%, 50% and 100%; 0% means no material extract and 100% means pure material extract). Extracts were added to cells in a defined volume per well (1 mL). Effects were analysed after 24 h and 72 h. Effects on cell metabolism were analysed with an in‐house MTT assay, cytotoxic effects with a lactate dehydrogenase assay (LDH) and a live‐dead staining (fluorescein diacetate FDA/propium iodide PI stain; see ‘Effects of pulp capping materials on cell viability and osteogenic differentiation’ section) was performed. As for the control group, cells without contact with extracts were used.

### Effects of pulp capping materials on cell viability and osteogenic differentiation

One hundred eighty test bodies of each material were produced as described above. The main study design as described above (approaches A, B and C; see Table 2) was performed. Effects on cell viability were analysed after 48 h (direct effects), 7 days (medium‐term effects) and 14 days (long‐term effects). Effects on proliferation were analysed with an in‐house MTT assay, cytotoxic effects with an LDH assay and a live‐dead staining was performed. As a control group, cells without contact to test bodies or extracts were used. Additionally, to analyse the effects on osteogenic potential, cells were cultivated under mineralisation conditions. After 14 days, effects on osteogenic differentiation potential were analysed with an alkaline phosphatase assay (ALP) (abcam, Cambridge, UK). Mineralisation potential was analysed with a modified alizarin red S quantification assay (ScienCell Research Laboratories, Carlsbad, CA, USA).

For the assessment of cell viability, an in‐house MTT assay was used to estimate cell metabolism as previously described (Sabandal et al., 2020). The cellular NAD(P) reflux converts the yellow thiazolyl blue tetrazolium bromide (0.5 mg/mL) (Merck/Sigma‐Aldrich, Darmstadt, Germany) into the violet formazan dye, which is measured photometrically at a 570 nm wavelength. Cytotoxic effects were measured with the CyQuant™ LDH Assay (Invitrogen, Life Technologies GmbH, Darmstadt, Germany). All assays were performed according to the manufacturers' protocols. Colorimetric determination was done with the Epoch microplate spectrophotometer (BioTek, Winooski, VT, USA). For the LDH assay, the factor related to control is shown. The qualitative analysis of cell viability was performed with a live‐dead staining using fluorescein diacetate (FDA)/propidium iodide (PI). Viable cells were stained green by FDA (Sigma‐Aldrich, St. Louis, MO, USA) and the nuclei of necrotic and apoptotic cells were stained red by PI (all Merck/Sigma‐Aldrich, Darmstadt, Germany).

The osteogenic potential of the human dental pulp cells was measured by alkaline phosphatase (ALP) activity. Cells were lysed with the Pierce IP Lysis Buffer (Life Technologies GmbH, Darmstadt, Germany). The supernatant was frozen at −80°C for subsequent assays. The Pierce BCA Protein Assay (Life Technologies GmbH, Darmstadt, Germany) was used for protein quantification. The Alkaline Phosphatase Assay Kit (abcam, Cambridge, UK) was used for the detection of alkaline phosphatase activity. Colorimetric determination was done with the Epoch microplate spectrophotometer (BioTek, Winooski, VT, USA). The percentage of ALP related to whole protein is shown. The capability of mineralisation was analysed with a modified alizarin red S staining (Alizarin Red S Staining Quantification Assay; ScienCell Research Laboratories, Carlsbad, CA, USA) as previously described (Sabandal et al., 2020). After fixation with formaldehyde (4% in phosphate‐buffered saline), cells were stained with an alizarin red S solution (40 nM, pH 4.1). Staining was dissolved in 10% acetic acid. The dissolved staining solution was heated to 85°C for 10 min and centrifuged for 10 min at 20 000×*g* before quantification. The supernatant was neutralised with a 10% ammonia solution (all Merck/Sigma‐Aldrich, Darmstadt, Germany). For quantification, a standard row of alizarin red S was carried out. The dissolved alizarin red S was measured at a wavelength of 405 nm. Colorimetric determination was done with the Epoch microplate spectrophotometer (BioTek, Winooski, VT, USA).

### Statistical analysis

Statistical analysis was carried out by one‐way anova using a modified Levene testing and *p* < 0.05, as well as a post hoc analysis with Tukey testing and the two‐sample T‐test to double‐check the results (Daniel's XL Toolbox version 6.53, http://xltoolbox.sourceforge.net (Kraus, 2004) and RStudio, Posit PBC [formerly RStudio Inc.], Boston, MA, USA).

## RESULTS

### Release of calcium ions

**FIGURE 1:**
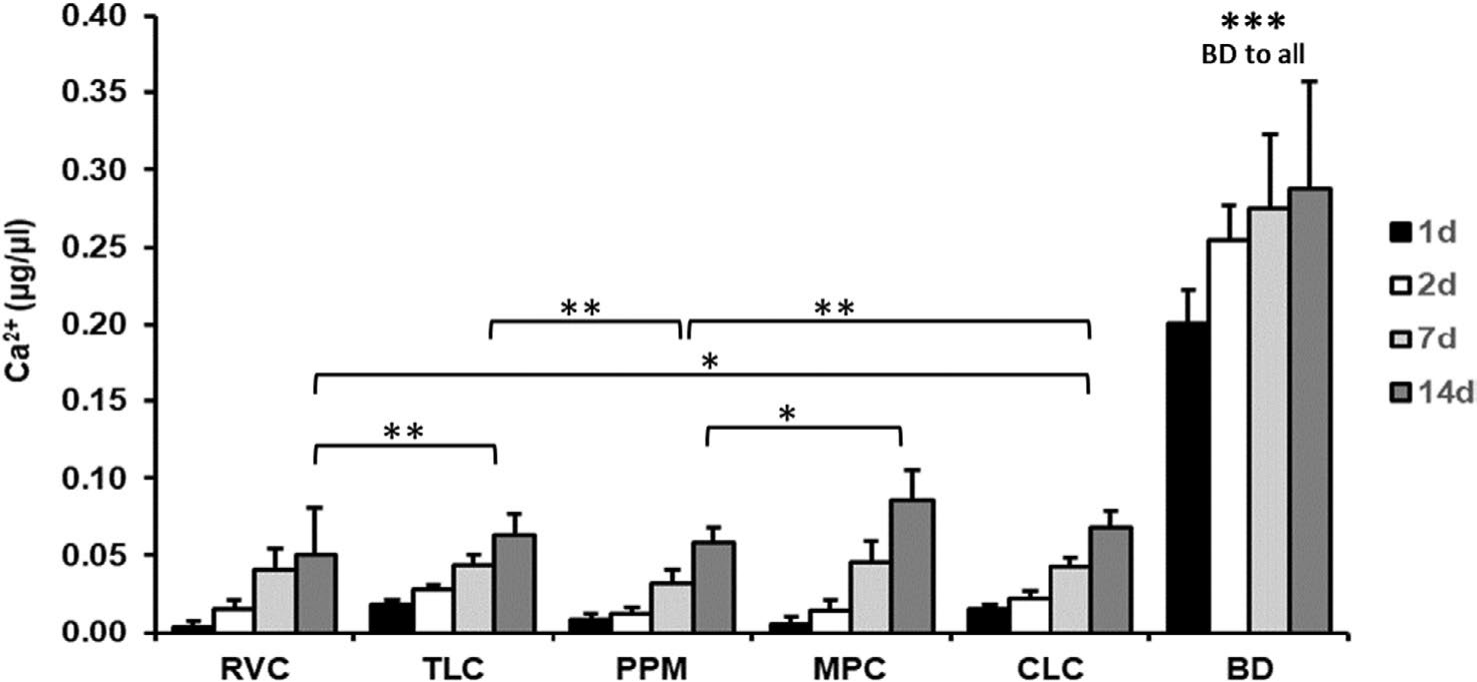
Release of calcium ions in μg/μL from the pulp capping materials after 1, 2, 7 and 14 days (release of calcium ions is shown in relation to control group; statistically significant differences between groups are marked by asterisks; ***very strong significance, **strong significance, *significance; BD showed highly statistically significant differences to all materials).

All LCPCM released calcium ions to the medium in a comparable concentration (Figure 1). Statistical analysis showed that the difference between BD and the light‐cured pulp capping materials was statistically significant at all time intervals, as well as the difference between RVC and TLC, RVC and CLC, PPM and MPC, PPM and CLC, as well as TLC and CLC (*p* < 0.05) (Figure 1).

### Effects of pulp capping materials' extracts on cell viability in different dilutions

**FIGURE 2:**
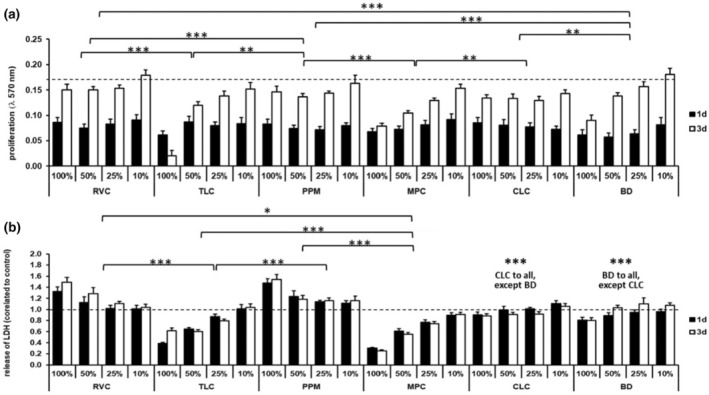
Effects of pulp capping material extract in 10%–100% dilution on cell viability after 1 and 3 days. (a) shows changes in proliferation (dashed line marked proliferation level of control group at day 1 of 0.17 in mean, proliferation at 3 days was 0.35 in mean; statistical significances between groups are marked by asterisk). (b) shows LDH release related to the control group (dashed line marked control rate; statistical significances between groups are marked by asterisk; ***very strong significance, **strong significance, *significance); control group corresponds to 0% material extract.

The mean proliferation for the control group was 0.17 (±0.010) at Day 1 and 0.35 (±0.014) at Day 3 (data not shown in Figure 2). Only with 10% extract, the level of proliferation in the control group at Day 1 could be reached at Day 3 (Figure 2, dashed line in Figure 2a). A statistical analysis was performed; the results are described below and presented in Figure 2.

For TLC and MPC, concentration‐dependent effects on cell viability are measurable. The proliferation rate decreased with increasing extract concentration (Figure 2a). No corresponding LDH release is observed (Figure 2b). For RVC and PPM, only slight effects on cell proliferation are observable. Until a concentration of 25% extract, changes in proliferation are similar to each other; just with 10% extract, a rise in proliferation rate is shown (Figure 2a). The corresponding LDH release indicates concentration‐dependent effects. For both materials, the LDH release decreases with decreasing concentration rate and is comparable with the control group with 10% extract (Figure 2b). CLC showed similar effects on proliferation rate or LDH release in all tested extract concentrations (Figure 2b). BD showed a concentration‐dependent effect on the proliferation rate and no significant LDH release was observable (Figure 2). Statistical analysis was performed; the results are marked in Figure 2a,b by asterisks. For the MTT assay, statistically significant differences between RVC and TLC, RVC and PPM, TLC and PPM, PPM and MPC, MPC and CLC, BD and CLC, BD and PPM and BD and RVC could be shown (*p* < 0.05) (see Figure 2a). For the LDH assay, statistically significant differences between BD and all other tested materials, as well as CLC and all other tested materials and between RVC and TLC, RVC and MPC, TLC and PPM, TLC and MPC and PPM and MPC were observed (*p* < 0.05) (see Figure 2b).

### Effects of pulp capping materials on cell viability and osteogenic differentiation

#### Cell viability

**FIGURE 3:**
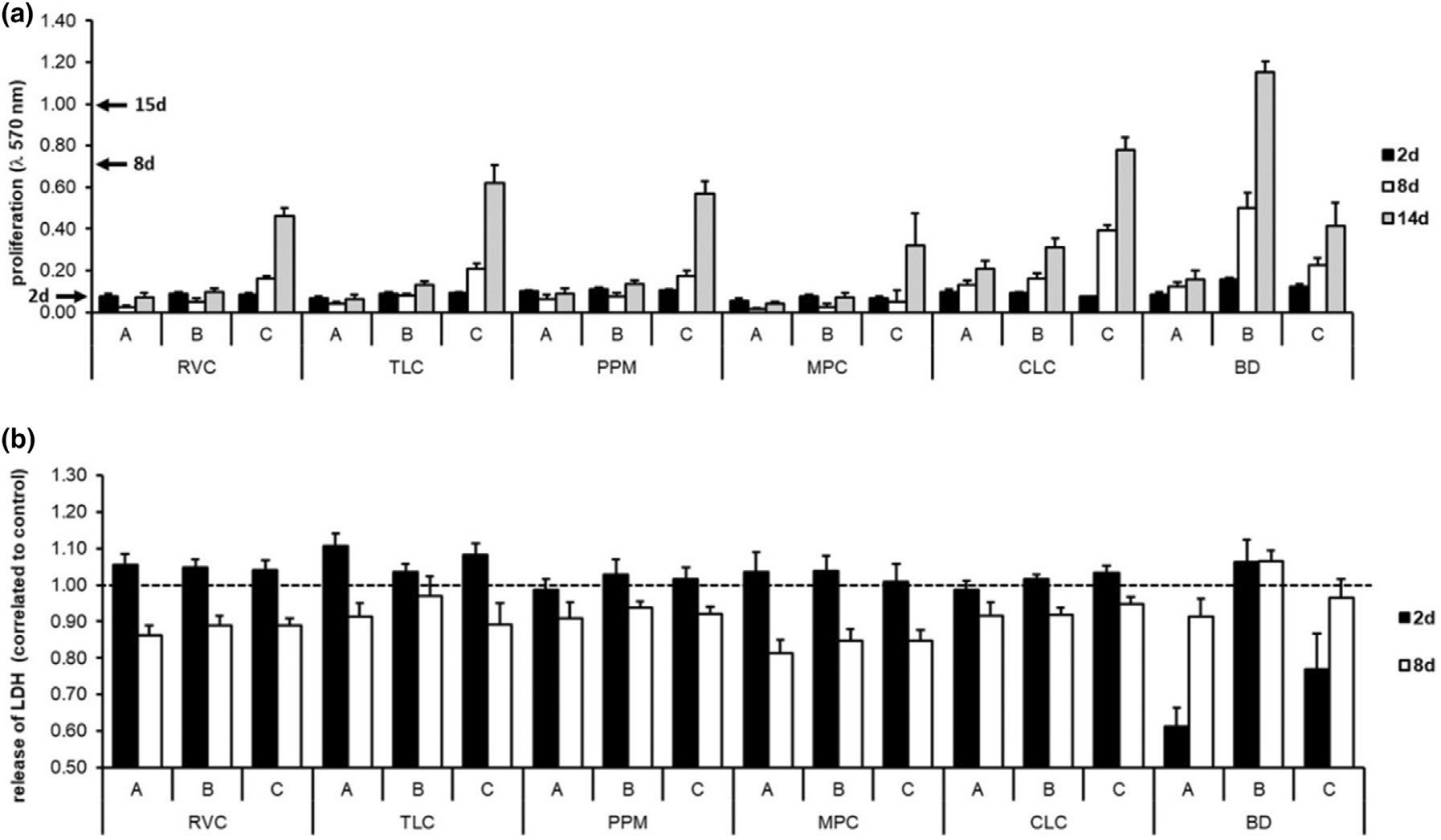
Effects of pulp capping material on survival of human dental pulp cells in approach A, B and C (see Pulp capping materials and main study design). (a) shows changes in proliferation (data for control group shown as arrows in mean). (b) shows LDH release related to control group (dashed line marked control rate).

**FIGURE 4:**
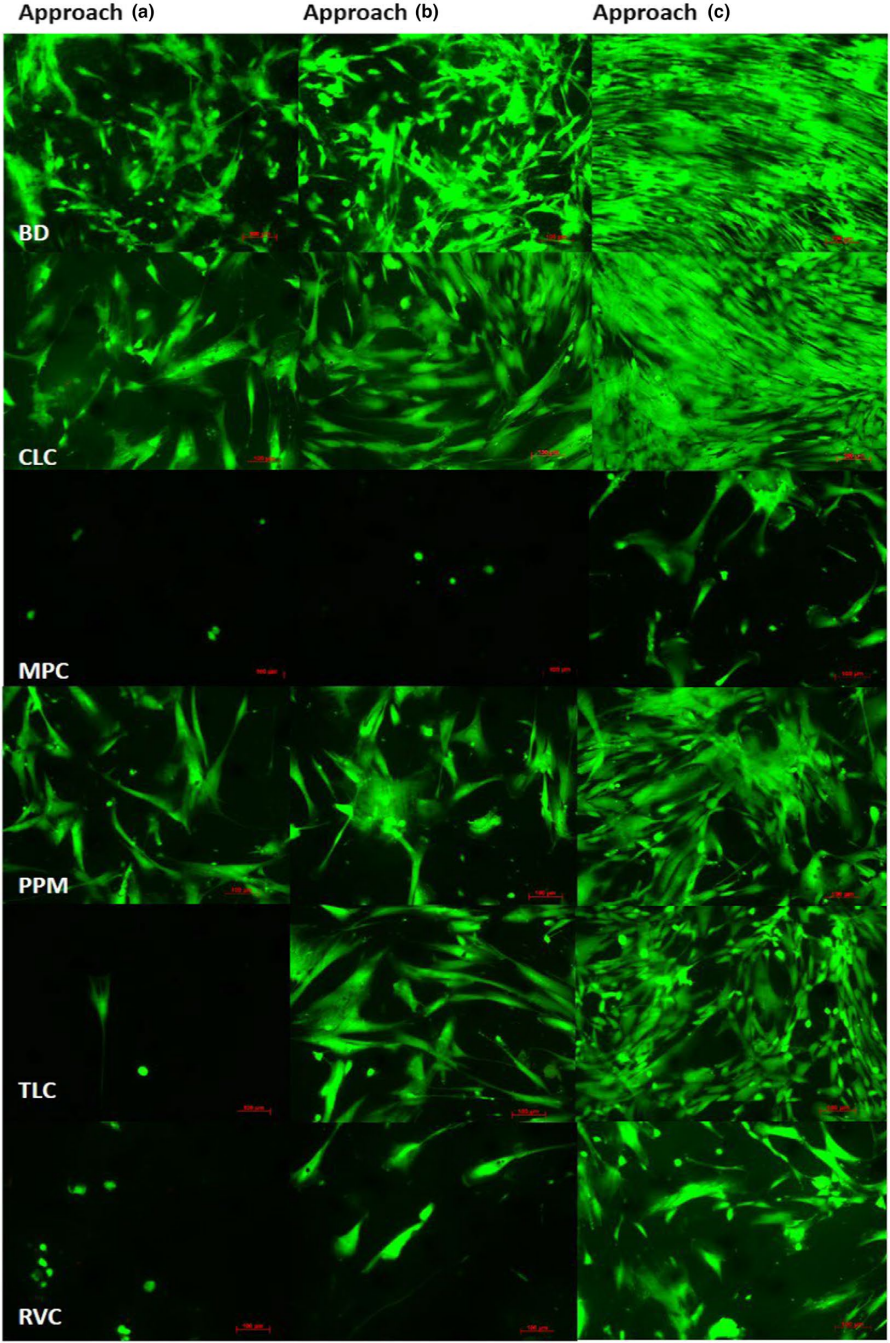
Live‐Dead staining of human dental pulp cells with pulp capping materials in approaches a, b and c (see Pulp capping materials and main study design) at day 8 (staining for control groups are not shown. magnification ×100, red scale bar = 100 μm).

The evaluation of the long‐time survival of human dental pulp cells revealed differences between the tested materials. Disregarding CLC and BD, cells in contact with MPC, PPM, RVC or TLC were dead or survived with a minor cell viability in approaches A and B. Only a slight cell regeneration during time is observable (Figure 3a). No release of LDH was observable (Figure 3b). When the test bodies remained in the wells (approach A and approach B), RVC, MPC and TLC kill the cells and, in the case of TLC, a regeneration was observable in approach B (Figure 4). By light microscopy (images shown in Figure S1), no cells or cells with altered morphology are visible near the test bodies. In contrast, cells survive and a regeneration over time is observable in approach C. The differences are not statistically significant (*p* > 0.05). For CLC, cells survive in all three approaches. In approaches A and B, the proliferation rate is lower compared to approach C; the differences between the approaches are not statistically significant (*p* > 0.05). For BD, cells survive in all approaches and regeneration over time is observable. The proliferation rate in approach B is higher compared to control groups; the difference is not statistically significant (*p* > 0.05). Furthermore, it appeared that cells mineralise in all BD approaches. The differences in the proliferation rates and the LDH release reported were not statistically significant (*p* > 0.05).

#### Osteogenic potential and mineralisation

**FIGURE 5:**
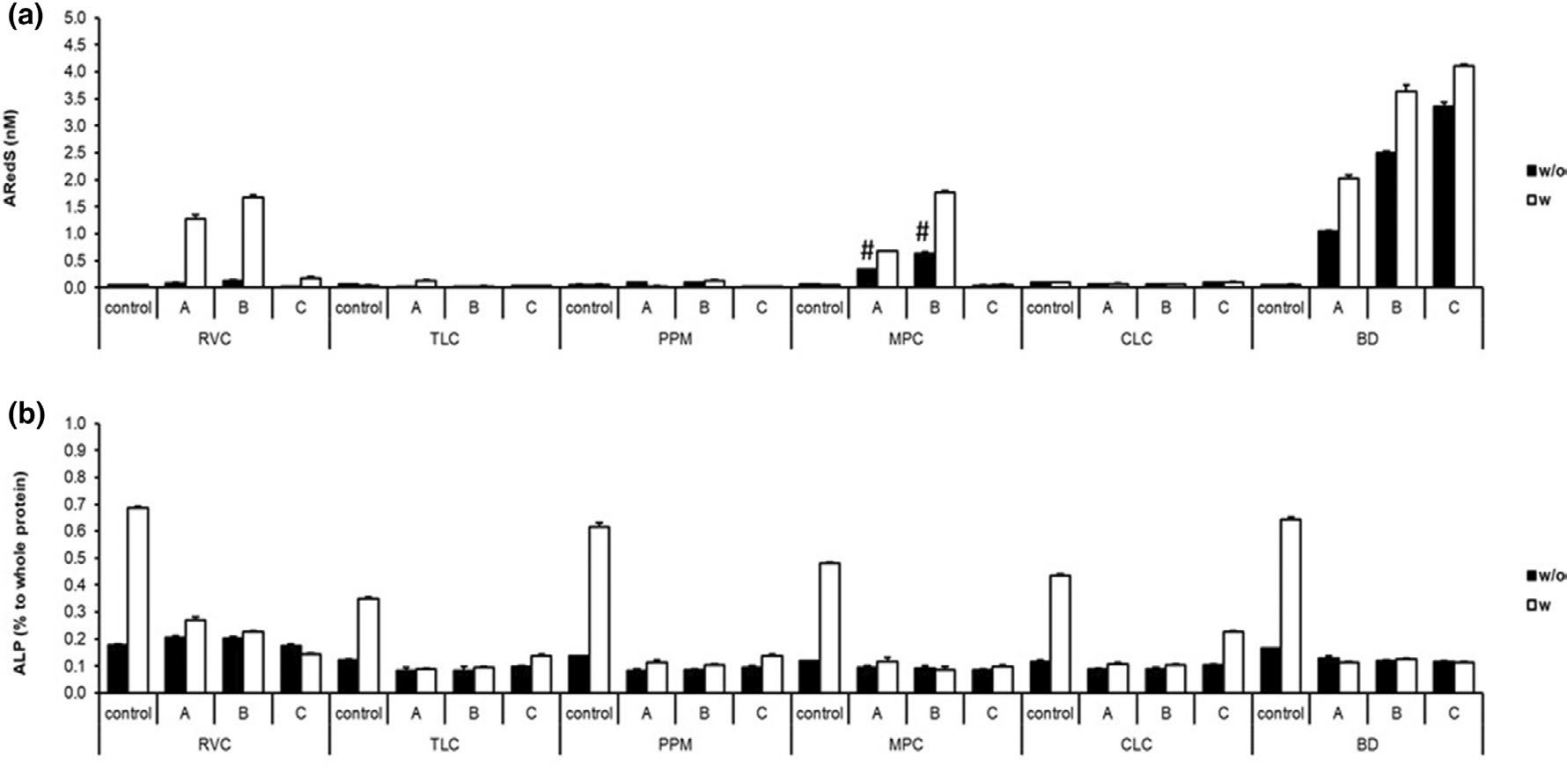
Effects on osteogenic and mineralisation potential of human dental pulp cells by capping material in approaches A, B and C (see Pulp capping materials and main study design). (a) shows the effects onto mineralisation potential by an alizarin red s (ARedS) assay after 14 days without (w/o) and with (w) mineralisation inducing medium (# ARedS staining comes from the material itself). (b) shows effects onto osteogenic potential by an alkaline phosphatase activity (% of ALP to whole protein) after 14 days without (w/o) and with (w) mineralisation inducing medium.

All three self‐isolated human dental pulp cell lines mineralise with BD. First hints of an initial mineralisation appear within the first 3–5 days, visible under the light microscope. The mineralisation rate was higher under mineralisation‐inducing conditions (Figure 5a). With RVC and MPC, in approaches A and B, cells mineralise under mineralisation‐inducing conditions only. For TLC, PPM and CLC, no mineralisation was found, neither with nor without mineralisation‐inducing conditions. No control group mineralises within the first 14 days (Figure 5a). The results from the ALP assay do not confirm the findings described above. In control groups only, ALP levels increase under mineralisation‐inducing conditions (Figure 5b). Just RVC showed a slight increase in approaches A and B, thus confirming the observed mineralisation. The ALP level in all approaches and culturing conditions with BD remains constant. The material itself leads to mineralisation and does not activate the mineralisation, according to all results for BD. The differences between the materials were not statistically significant (*p* > 0.05).

## DISCUSSION

The present study was conducted on three cell lines of human dental pulp cells harvested from three different patients. The possible cytotoxic effects as well as the ability to induce mineralisation of five LCPCM were compared to Biodentine. The results of the present study can be classified as reliable, as all three cell lines showed a similar behaviour towards the tested materials. In addition, the test bodies were all fabricated with glass plate coverage on both sides to prevent the formation of an oxygen inhibition layer on the surface of the LCPCM and the manufacturers' instructions were strictly followed.

This study assessed the effects of the investigated materials on cell viability and cell mineralisation as well as the capacity of the materials to release calcium ions. The effects on gene expression or cell apoptosis caused by the materials were not investigated, which can be considered a limitation of the present study and should be included in further studies. But for the very first time, the LCPCM ReViCal, MTA PulpCap and Pulprotec MTA were compared to the HCSC Biodentine in terms of biocompatibility and their effects on human dental pulp cells.

Another limitation is the in vitro setting of the study. The effects of LCPCM were only investigated on a single cell type and not on the entire pulp tissue. However, even if other pulp cells could tolerate contact with LCPCM, this would not speak in favour of LCPCM in VPT, as the hDPC were demonstrably damaged in this study.

The results indicate that the LCPCM are inferior to Biodentine regarding their biological properties. They have significantly higher cytotoxic effects on hDPC regardless of their concentration. They also trigger the death of the hDPC, which results in lower viability. Biodentine only caused mechanical irritation to the cells because of the debris in the wells and induced early mineralisation. Strikingly, the expression of LDH of hDPC in contact with the LCPCM roughly corresponds to the LDH levels of the control group; however, in the MTT assay, less cell metabolism was detectable. This could point towards apoptosis of hDPC. If, due to contact with the material, the cells become apoptotic, no elevated LDH levels can be detected. In future studies, an additional annexin assay should be conducted to undoubtedly prove cell apoptosis.

hDPC in contact with Biodentine mineralised in all approaches and even without the mineralisation‐inducing medium. Biodentine is the only one of the investigated materials that is able to induce cell mineralisation of hDPC without the addition of mineralisation inducing medium. Yet it is surprising that in the present study no induction of the osteogenic potential of hDPC through Biodentine could be ascertained, as no elevated ALP levels of hDPC in contact with Biodentine were found. In the literature, studies which attest to an induction of the osteogenic potential of cells on a genetic level through HCSC can be found (Rodríguez‐Lozano et al., 2021), in future studies, investigations of gene expression in contact with LCPCM or HCSC should be included.

In the relevant literature, there are studies that have proven that pulpal stem cells in contact with TheraCal LC produce less dentin‐sialoprotein (Jeanneau et al., 2017). In teeth capped with TheraCal LC, osteocalcin and dentin‐sialoprotein were lacking (Lee et al., 2015), both of which are essential for the mineralisation process. This points towards a potential reason why LCPCM are not able to induce cell mineralisation. LCPCM release cytotoxic monomers into the surrounding medium, thus damaging the cells and inhibiting physiological cell function. Proteins essential for the mineralisation process are barely expressed when cells are in contact with LCPCM; as a result, mineralisation cannot take place.

The only material property investigated in the present study is the materials' ability to release calcium ions into the cell culture medium. It is noticeable that Biodentine is significantly superior to the LCPCM, as it releases three times more calcium ions. These findings correspond to other studies that can be found in the relevant literature. When stored in distilled water or simulated body fluids, LCPCM release less calcium ions into the storage solution than calcium silicate cements (Arias‐Moliz et al., 2017; Camilleri, 2014; Gandolfi et al., 2015). Compared to HCSC, composite resins set through a polymerisation reaction. If the polymerisation reaction is not complete, the composite resins release more toxic monomers (Issa et al., 2004; Nilsen et al., 2017) than in a completely polymerised state (Aranha et al., 2010; Jontell et al., 1995). If one considers that an incomplete polymerisation leads to an increased cytotoxicity of composite materials, the results of the present study are all the more remarkable. By using glass plates, the test bodies were free from any oxygen inhibition layer on their surfaces. Furthermore, they were completely polymerised taking into account the maximum layer thickness according to the manufacturers' instructions. But still, the LCPCM exhibited negative effects on cell viability.

TheraCal LC is the most investigated in the relevant literature, followed by Calcimol LC. However, little or even no information is available on the other materials used in this study. The results of these LCPCM are therefore of particular interest. TheraCal LC reduces the proliferation rate of pulpal cells (Hebling et al., 2009; Poggio et al., 2015) and has a significant cell‐damaging effect when in direct contact with human pulpal cells (Bortoluzzi et al., 2015; Giraud et al., 2018, 2019; Hebling et al., 2009;Poggio et al., 2014; Poggio et al., 2015). A significantly elevated cytotoxicity of TheraCal LC and Calcimol LC was reported in comparison to Biodentine and MTA (Poggio et al., 2014, 2015). Other comparisons between TheraCal LC and Biodentine or MTA also revealed that TheraCal LC is more cytotoxic than the calcium silicate cements (Küden et al., 2022; Rodríguez‐Lozano et al., 2021; Sanz et al., 2021). These findings correspond to the findings in the present study.

Compared to HCSC, LCPCM also lack the ability to induce cell mineralisation (Manaspon et al., 2021) or exhibit a far lesser ability to do so (Bortoluzzi et al., 2015; Sanz et al., 2021). This corresponds to the results of the present study, which also indicate that the LCPCM lack the ability to induce the mineralisation of hDPC.

In histological examinations of the pulp, it was proven that even though the indirectly or directly pulp capped teeth were clinically symptom‐free, an inflammatory reaction of the pulp and a reduced hard tissue formation for the teeth capped with TheraCal LC was found, while in the pulps capped with Biodentine or MTA, the odontoblast layer appeared normal and no inflammatory reaction of the pulp was detectable (Bakhtiar et al., 2017; Lee et al., 2015; Sahin et al., 2021). These results highlight that even though in‐vivo studies comparing LCPCM to calcium silicate cements are scarce, the studies assessing the clinical outcome of pulp capping as well as their histological outcome conclude that LCPCM are less biocompatible than calcium silicate cements.

In summary, out of five investigated LCPCM, three were examined for the first time (ReViCal, MTA PulpCap, Pulprotec MTA). Though hDPC exhibited slightly differing behaviour towards the LCPCM used here, they all have in common that they have cytotoxic effects on hDPC and hardly induce cell mineralisation. ReViCal and MTA PulpCap show a similar behaviour to TheraCal LC; Pulprotec MTA is a little less cytotoxic than the others (difference not statistically significant), but none of them is able to induce cell mineralisation. Taking into consideration the results of the present study and the available relevant literature, the suitability of light‐cured pulp capping materials for VPT needs to be reassessed.

## CONCLUSION

The results of the present in vitro study strongly indicate that light‐cured pulp capping materials are significantly more cytotoxic than Biodentine when exposed to human dental pulp cells and lack the ability to induce mineralisation of human dental pulp cells. Thus, they can be classified as inferior to calcium silicate cements in vitro. The application and suitability of LCPCM for use in vital pulp therapy when compared to hydraulic calcium silicate cements should be reconsidered.

## AUTHOR CONTRIBUTIONS

Laurentia Schuster conceptualization (equal), formal analysis (lead), funding acquisition (lead), investigation, project administration, writing—original draft preparation (lead); Sonja Sielker conceptualization (equal), funding acquisition, investigation (lead), project administration (lead), writing—original draft preparation; Johannes Kleinheinz resources (lead); Till Dammaschke conceptualization (equal), funding acquisition, project administration, supervision (lead), writing—review and editing (lead).

## FUNDING INFORMATION

The study was funded by the DGR^2^Z‐GC‐Grant research funding.

## CONFLICT OF INTEREST STATEMENT

The authors declare no conflict of interest. Till Dammaschke has received fees from Septodont for lectures. Laurentia Schuster has received a research grant from the Deutsche Gesellschaft für Restaurative und Regenerative Zahnerhaltung (DGR^2^Z).

## ETHICS STATEMENT

The Ethical Committee of the University of Münster (Germany) has given its approval (# 2022‐432‐f‐S).

## Supporting information


**Figure S1.** Light‐microscope images of human odontoblast cells with pulp capping materials in approaches A, B and C (see Pulp capping materials and main study design) at day 14 (the black parts in approach A and B show the test bodies; images for control groups are not shown; magnification ×100, black scale bar = 100 μm).

## Data Availability

The data that support the findings of this study are available from the corresponding author upon reasonable request.
