# Supplementary figures and images for: Effect of light‐cured pulp capping materials on human dental pulp cells in vitro
Source: Int Endod J. 2025 Apr 25;58(7):1060–72. doi: 10.1111/iej.14242 (PMC12160968; doi:10.1111/iej.14242)

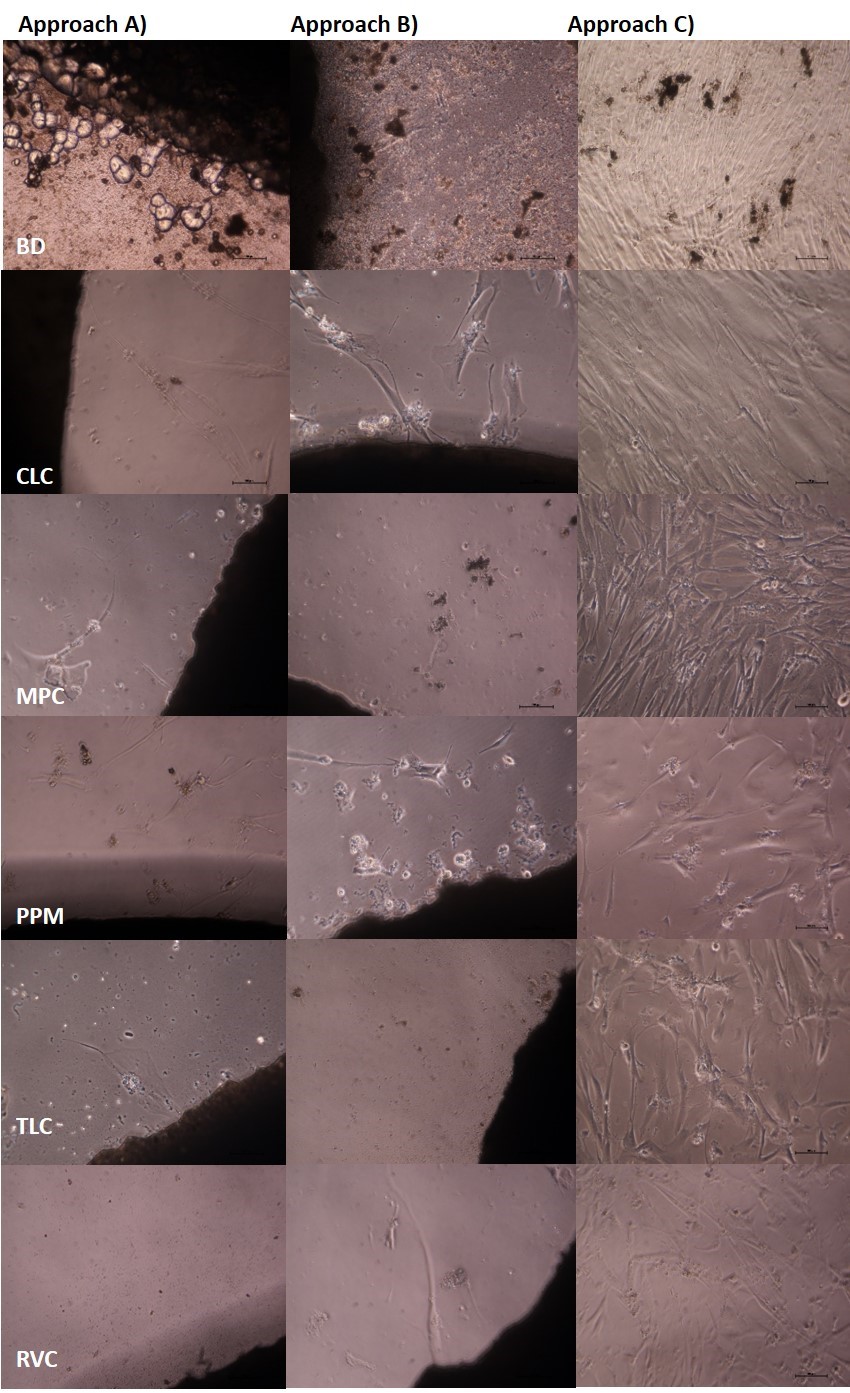

Supplement: Supplementary file 1 — Figure S1. Light‐microscope images of human odontoblast cells with pulp capping materials in approaches A, B and C (see Pulp capping materials and main study design) at day 14 (the black parts in approach A and B show the test bodies; images for control groups are not shown; magnification ×100, black scale bar = 100 μm). [file IEJ-58-1060-s001.jpg]
